# Angiofibroma of the Mandible: Report of a Rare Case

**DOI:** 10.1155/2022/7779338

**Published:** 2022-04-05

**Authors:** Mohammed A. AlZayer, Ali Matouq AlMarzouq, Zahrah Hasan Al-Faraj, Eman F. Al-Saleh

**Affiliations:** ^1^Dammam Medical Complex, Saudi Arabia; ^2^Qatif Central Hospital, Saudi Arabia; ^3^Dammam regional lab and blood bank, Saudi Arabia

## Abstract

Angiofibroma is an uncommon, highly vascular benign lesion that occurs in the head and neck region, typically arising in the nasopharyngeal area, potentially with a locally aggressive course. Angiofibroma with a primary intraoral presentation is extremely rare; few case reports have been published in the literature, with only three cases of angiofibroma in the mandible published to date. In this case, a 37-year-old man presented with swelling at the right mandible and underwent enucleation of the lesion under general anesthesia. After 1-year follow-up, there were no signs of recurrence.

## 1. Introduction

Angiofibroma is a rare, benign, highly vascularized tumor that commonly presents during male adolescence. Its origin is unknown [1]. It typically arises in the nasopharyngeal area but can develop in extrapharyngeal sites, the most common of which is the maxillary sinus [2]. Although angiofibroma is histologically a benign lesion, it is locally invasive and has a propensity for local extension into adjacent anatomic structures, including the oral cavity [3]. Few cases have been published reporting angiofibroma with a primary intraoral origin [1, 4, 5], with only three cases of angiofibroma in the mandible published to date [4, 6, 7].

## 2. Presentation of Case

**Figure 1:**
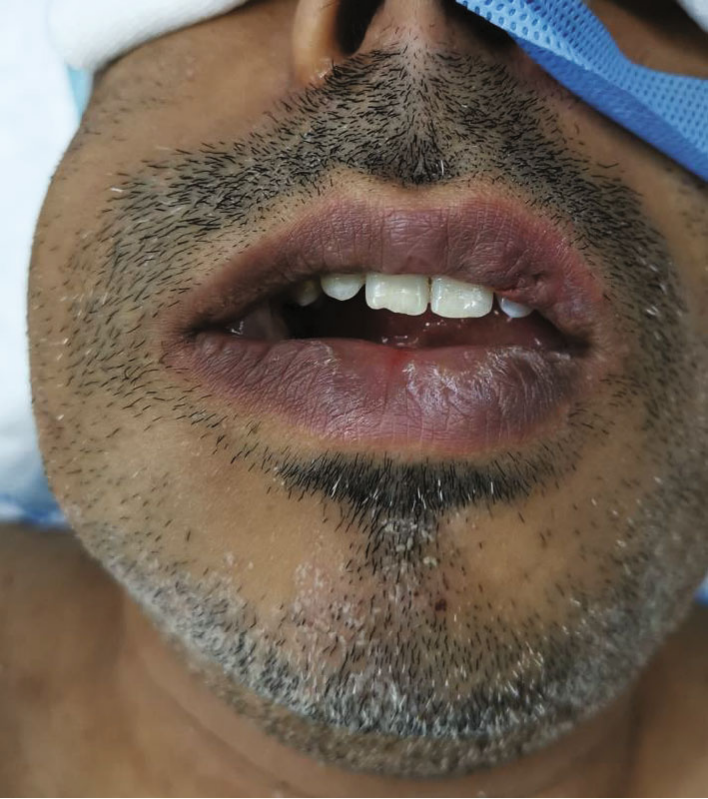
Extraoral photograph of the patient showing swelling on right side of the face.

The patient (37 years old; male) presented with swelling of the right posterior mandibular area. He had a clear medical history. He complained of a small, dark blue-colored localized swelling at the area of the attached gingiva between teeth numbers 45 and 46 for 24 days before he went to a general dentist. An antibiotic was prescribed for five days, but his condition did not improve, so the dentist decided to extract tooth number 46. After extraction, the swelling started to gradually increase until it occupied the buccal vestibule. He noted pus and bleeding from the mass. Extraoral examination revealed significant swelling on the right side of the face that extended from the inferior border of the mandible to the level of the zygoma (Figure 1). Swelling was soft and fluctuant; no drainage or change in skin color was noted. All facial nerve branches were functioning well.

**Figure 2:**
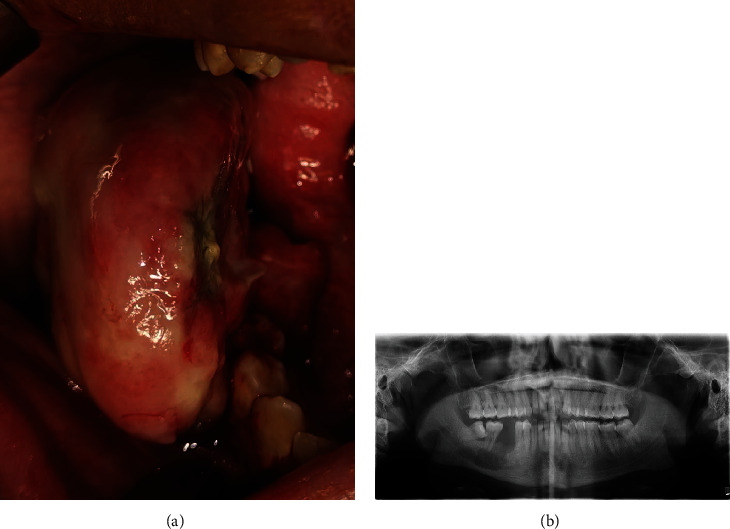
(a) Intraorally, the lesion obliterated the right buccal vestibule. (b) Orthopantogram revealed a unilocular radiolucency at the right mandible.

The patient had no history of paresthesia related to the inferior alveolar or lingual nerve. Intraorally, a huge mass was found to occupy the right buccal vestibular area. It was firm, measured 5 × 5 × 3 cm, red in color, covered with a whitish film, and pedunculated from the extraction socket, with ulceration noted at the level of the occlusal table due to mastication (Figure 2(a)). Orthopantogram revealed a unilocular radiolucency at the right mandible, periapical to the lower right first and second molar area (3 cm in the anterior-posterior dimension) (Figure 2(b)).

Incisional biopsy was performed, and the extraosseous portion was excised and sent for histopathological assessment. Bleeding was encountered during the biopsy, which was controlled by local measures (Surgicel and suture). At the 1-week follow-up visit, the mass had grown again and reached its previous size.

**Figure 3:**
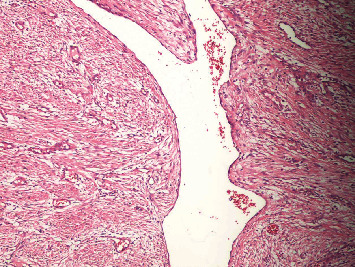
Hematoxylin and eosin stain (H&E) showing the proliferation of fibrous tissue with abundant variably sized vascular channels interspersed among the spindle cells in background, consistent with the diagnosis of angiofibroma (10x).

Histopathological findings were suggestive of angiofibroma. Microscopy revealed an ulcerated, markedly inflamed squamous epithelial mucosa with underlying proliferation of fibrous tissue with interspersed abundant vascular channels. The vascular channels had architectural variability from the small capillaries to the sinusoidal ectatic vessels, to the larger vessels of venous type with distinct muscle layer. The fibrous stroma consisted of abundant collagen fibers of variable density and stellate or ovoid spindle cells with a fibroblastic/myofibroblastic appearance (Figure 3).

Computed tomography (CT) angiography was performed to assess lesion vascularity. No obvious large vascular channel supplying the lesion was detected. A large venous tributary in the vicinity of the lesion was observed to be draining into the right external jugular vein. A bony perforation at the lingual side was associated with enhanced soft tissue component.

**Figure 4:**
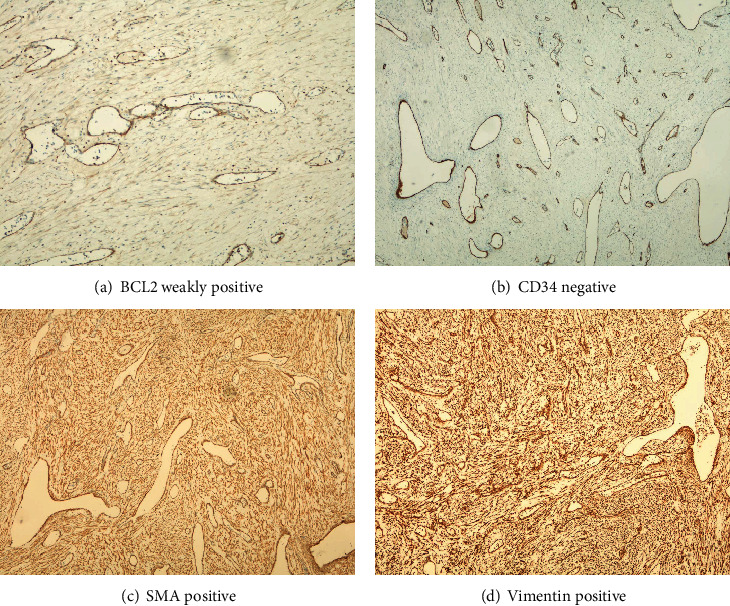
Immunohistochemical analysis using different stains.

The decision was made to perform enucleation of the lesion under general anesthesia. The patient was in the supine position during the operation and received oral intubation; hypotensive general anesthesia was applied. Excision of the extraosseous part was performed along with enucleation of the intraosseous lesion. The surgery was uneventful, and the lesion was easily detached from the bone with no adhesion noted. Encountered bleeding was controlled using cautery and bone wax. The wound was closed primarily. The final histopathological report confirmed the presence of angiofibroma and provided the same histopathological description. Immunohistochemical analysis using different stains has been performed to confirm the diagnosis. The lesional cells were positive for vimentin, smooth muscle actin, and Bcl2 and negative for Ck AE1/AE3, desmin, CD34, and S100Prot (Figures 4(a)–4(d)).

**Figure 5:**
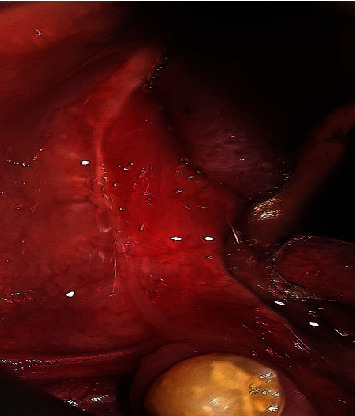
One-year postoperative intraoral photograph.

The patient received regular follow-up (1 week, 2 weeks, 1 month, 3 months, 6 months, and 1 year). Healing was within normal limits, and no recurrence of overgrowth, swelling, or discharge has been observed (Figure 5).

**Figure 6:**
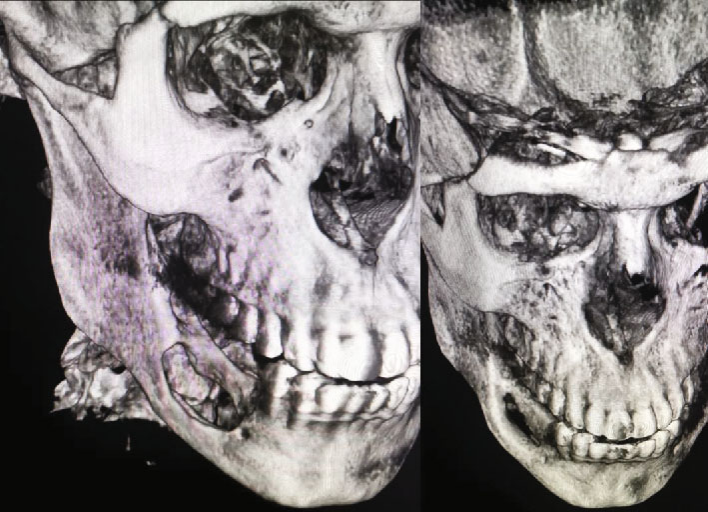
Preoperative and one-year postoperative 3D CT scan.

At 1-year follow-up, CT with contrast and CT angiogram were performed to confirm no recurrence and no arterial or venous regrowth. The report concluded the presence of bone defect with no soft tissue component and no abnormal arterial or venous enhancement. Comparing the preoperative CT with the 1-year postoperative CT, bone regeneration was noted, and only a small portion of the inferior border defect remained (Figure 6).

## 3. Discussion

The significance of this report lies in the rarity of the presence of angiofibroma, which commonly occurs in the nasopharynx, in the mandible.

The etiology of angiofibroma is unclear. Theories about the origin and histogenesis of angiofibroma vary (i.e., developmental, hormonal imbalance, and genetic causes) [8].

Angiofibroma are rare tumors of the maxillofacial region, accounting for 0.05% of head and neck tumors [9]. The presence of angiofibroma in the mandible is extremely rare; only a few cases have been published reporting the presence of angiofibroma in the lower jaw (Table 1) [4, 6, 7].

It is suspected that ectopic tissue may be located farther away from the usual place and may have been the cause of the extranasopharyngeal location [8]. Histologically, tumor has a basic characteristic pattern and is derived from two equally important components: vascular network and connective tissue stroma. Vessels typically vary in size and are irregularly shaped [10].

Nasopharyngeal angiofibromas in the head and neck area usually arise in adolescent males [11]. In their literature review, Ali and Jones compared nasopharyngeal angiofibroma incidence with extrapharyngeal angiofibroma between patients of different sex and age distributions and found that nasopharyngeal angiofibroma is correlated with both age and sex. That is, they found that tumors are seen almost exclusively in males, and their appearance during the second decade of life is one of their most characteristic features. However, they also reported that extranasopharyngeal angiofibromas do not follow the same characteristic age distribution and sex incidence of nasopharyngeal angiofibromas [12]. In 2004, Windfuhr and Remmert evaluated extrapharyngeal angiofibroma incidence and found that patients were older as compared with patient with nasopharyngeal angiofibroma [11].

Depending on the size and location of the angiofibroma, different treatment modalities are available. Most physicians agree that surgery is the primary treatment modality for early-stage disease [13]. Preoperative angiography is the best tool for depicting the vascularization of juvenile angiofibroma, which predominantly receives its vascular supply via the external carotid artery system [14].

In our case, no embolization was planned, as there was no obvious large vascular channel supplying the lesion. No serious bleeding was encountered during the operation. Some authors have reported the successful surgical treatment of juvenile angiofibroma with acceptable blood loss without embolization [3, 5, 14].

Some researchers advised resection to prevent recurrence [5, 8]. Most recurrences produce symptoms within 1 year after treatment, and recurrence is uncommon beyond two years after surgery [15]. No recurrence was noted in our case after 1 year. A similar result has been reported in other studies [4, 6, 7].

Other treatment modalities include radiation therapy, cryotherapy, hormone therapy, embolization, arterial ligation, and sclerosing agent use [16]. May et al. recently reported the use of percutaneous cryoablation for an 18-year-old man who presented with a right condylar angiofibroma with complete recovery and no recurrence after 1-year follow-up [6].

## 4. Conclusion

The significance of this report lies in the rarity of the presence of angiofibroma, which commonly occurs in the nasopharynx at young age, in the mandible. Although exceedingly rare, angiofibroma should be considered one of the differential diagnoses of a lesion that arises in the mandible.

## Figures and Tables

**Table 1:** Comparing the previously published case with our case.

	Ul Khaliq et al. [4]	May et al. [6]	Amini-Salari et al. [7]	Our case
Age	16 years	18 years	23 years	37 years
Gender	Female	Male	Male	Male
Location	Right mandibular first premolar to the right mandibular third molar region	Right condyle	Left mandibular ramus	Right mandibular body
Size	Not mentioned	Not mentioned	1.8 cm × 0.7 cm × 4.1 cm	3 cm × 3 cm
Treatment	Surgical excision	Percutaneous cryoablation	Enucleation	Enucleation
Follow-up	1 year	1 year	6 months	1 year

## References

[1] Kabot T. E., Goldman M. E., Bergman S., Schwartz R. D. (1985). Juvenile nasopharyngeal angiofibroma: an unusual presentation in the oral cavity. *Oral Surgery, Oral Medicine, Oral Pathology*.

[2] Handa K. K., Kumar A., Singh M. K., Chhabra A. H. (2001). Extranasopharyngeal angiofibroma arising from the nasal septum. *International Journal of Pediatric Otorhinolaryngology*.

[3] Antoniades K., Antoniades D. Z., Antoniades V. (2002). Juvenile angiofibroma: report of a case with intraoral presentation. *Oral Surgery, Oral Medicine, Oral Pathology, Oral Radiology, and Endodontology*.

[4] Ul Khaliq M. I., Shah A. A., Dar N. (2016). A rare case of angiofibroma of the mandible: a case report. *Journal of Oral Biology and Craniofacial Research*.

[5] Capodiferro S., Favia G., Lacaita M. G., Lo Muzio L., Maiorano E. (2005). Juvenile angiofibroma: report of a case with primary intra-oral presentation. *Oral Oncology Extra*.

[6] May L., Blatter J., Bize P., Tsoumakidou G., Denys A., Broome M. (2020). Percutaneous cryoablation of benign bony tumours of the mandible. *British Journal of Oral and Maxillofacial Surgery*.

[7] Amini-Salari A., Glomski K., Ahn D., Tannyhill R. J. (2020). Mandibular intraosseous angiofibroma-a rare clinical entity. *Journal of Oral and Maxillofacial Surgery*.

[8] Akbas Y., Anadolu Y. (2003). Extranasopharyngeal angiofibroma of the head and neck in women. *American Journal of Otolaryngology*.

[9] Patterson C. N. (1973). Juvenile nasopharyngeal angiofibroma. *Otolaryngologic Clinics of North America*.

[10] Sternberg S. S. (1954). Pathology of juvenile nasopharyngeal angiofibroma; a lesion of adolescent males. *Cancer*.

[11] Windfuhr J. P., Remmert S. (2004). Extranasopharyngeal angiofibroma: etiology, incidence and management. *Acta Oto-Laryngologica*.

[12] Ali S., Jones W. I. (1982). Extranasopharyngeal angiofibromas. Sex incidence and age distribution. *The Journal of Laryngology & Otology*.

[13] Hodges J. M., McDevitt A. S., El-Sayed Ali A. I., Sebelik M. E. (2010). Juvenile nasopharyngeal angiofibroma: current treatment modalities and future considerations. *Indian Journal of Otolaryngology and Head & Neck Surgery*.

[14] Bertazzoni G., Schreiber A., Ferrari M., Nicolai P. (2019). Contemporary management of juvenile angiofibroma. *Current Opinion in Otolaryngology & Head & Neck Surgery*.

[15] Batsakis J. G. (1979). *Tumors of the Head and Neck: Clinical Pathological Considerations*.

[16] Jafek B. W., Nahum A. M., Butler R. M., Ward P. H. (1973). Surgical treatment of juvenile nasopharyngeal angiofibroma. *The Laryngoscope*.

